# DNAJC1 facilitates glioblastoma progression by promoting extracellular matrix reorganization and macrophage infiltration

**DOI:** 10.1007/s00432-024-05823-1

**Published:** 2024-06-22

**Authors:** Han Zhang, Wenjing Zheng, Xu Chen, Longqi Sa, Yi Huo, Lingling Zhang, Lequn Shan, Tao Wang

**Affiliations:** 1 State Key Laboratory of Holistic Integrative Management of Gastrointestinal Cancers, Department of Medical Genetics and Developmental Biology, Fourth Military Medical University, Xi’an, 710032 China; 2 Department of Spine Surgery, Honghui Hospital, Xi’an Jiaotong University, Xi’an, 710054 China; 3 State Key Laboratory of Holistic Integrative Management of Gastrointestinal Cancers, Department of Biochemistry and Molecular Biology, Fourth Military Medical University, Xi’an, 710032 China

**Keywords:** DNAJC1, Glioblastoma, Prognostic biomarker, Extracellular matrix reorganization, Macrophage infiltration

## Abstract

**Background:**

Glioblastoma (GBM) is a high-grade and heterogeneous subtype of glioma that presents a substantial challenge to human health, characterized by a poor prognosis and low survival rates. Despite its known involvement in regulating leukemia and melanoma, the function and mechanism of DNAJC1 in GBM remain poorly understood.

**Methods:**

Utilizing data from the TCGA, CGGA, and GEO databases, we investigated the expression pattern of DNAJC1 and its correlation with clinical characteristics in GBM specimens. Loss-of-function experiments were conducted to explore the impact of DNAJC1 on GBM cell lines, with co-culture experiments assessing macrophage infiltration and functional marker expression.

**Results:**

Our analysis demonstrated frequent overexpression of DNAJC1 in GBM, significantly associated with various clinical characteristics including WHO grade, IDH status, chromosome 1p/19q codeletion, and histological type. Moreover, Kaplan‒Meier and ROC analyses revealed DNAJC1 as a negative prognostic predictor and a promising diagnostic biomarker for GBM patients. Functional studies indicated that silencing DNAJC1 impeded cell proliferation and migration, induced cell cycle arrest, and enhanced apoptosis. Mechanistically, DNAJC1 was implicated in stimulating extracellular matrix reorganization, triggering the epithelial-mesenchymal transition (EMT) process, and initiating immunosuppressive macrophage infiltration.

**Conclusions:**

Our findings underscore the pivotal role of DNAJC1 in GBM pathogenesis, suggesting its potential as a diagnostic and therapeutic target for this challenging disease.

**Supplementary Information:**

The online version contains supplementary material available at 10.1007/s00432-024-05823-1.

## Introduction

Gliomas constitute over 70% of malignant brain tumors in the central nervous system (CNS) and represent the most prevalent primary brain tumors (Rong et al. 2022). The prognosis for glioma patients is bleak, with a 5-year survival rate of a mere 59%. Glioblastoma (GBM), the most aggressive and heterogeneous glioma subtype, exhibits a particularly dismal 2-year survival rate of 26% (Yaghi and Gilbert 2022). GBM is characterized by its rapid proliferation, invasiveness, and capacity to modulate the tumor microenvironment—factors that significantly contribute to its malignancy by impairing immune responses through the release of anti-inflammatory cytokines (Bellail et al. 2004). Additionally, the blood–brain barrier (BBB) poses a substantial obstacle to the delivery of therapeutics to GBM lesions (Yang et al. 2022). Despite advances in various treatment modalities, the median survival for GBM patients hovers between 14 and 17 months (Xie et al. 2020; Zhang et al. 2020). Recent research has implicated several genetic and epigenetic regulators in GBM pathogenesis; however, the central mechanisms remain elusive (Jia et al. 2016), necessitating the identification of effective targets to elucidate GBM’s underlying biology and to develop new treatment strategies.

Heat shock proteins (HSPs), a ubiquitous and evolutionarily conserved superfamily of proteins, are crucial molecular chaperones that safeguard the cell from various stresses and contribute to maintaining homeostasis (Nicchitta 2003; Kampinga and Bergink 2016; Aghdassi et al. 2007). Beyond their protective roles, HSPs also modulate an array of pathological states, including cancer (Dudeja et al. 2009). Their dysregulated expression in various tumor types implicates a significant role in cellular processes such as apoptosis, proliferation, differentiation, and immune modulation (Iglesia et al. 2019) (Nicchitta 2003). Nonetheless, the precise function and regulation of HSPs in GBM are not fully understood, although some have been identified as playing roles in tumor promotion or suppression (Rajesh et al. 2020).

The DnaJ Heat Shock Protein Family (HSP40) Member C1, also known as DNAJC1, is a highly conserved protein that assists in protein maturation and various cellular processes (Qiu et al. 2006). Abnormal expression levels of DNAJC1 homologs have been observed in gliomas, affecting tumor development and progression (Sun et al. 2020). Overexpression of DNAJC1 has been associated with tumor growth and invasiveness in leukemia and melanoma, suggesting a potential role in tumorigenesis (Papalas et al. 2010; Shiba et al. 2019). However, the exact functions and mechanisms of DNAJC1 in GBM remain to be elucidated.

This study employed bioinformatics techniques to analyze clinical characteristics, molecular markers, and immune cell infiltration in GBM, highlighting the prognostic and diagnostic potential of DNAJC1 as a clinical biomarker. In vitro analyses revealed DNAJC1’s role in promoting GBM cell proliferation, cell cycle progression, and migration. Moreover, the study uncovered that DNAJC1 facilitates epithelial-mesenchymal transition (EMT) and attracts immunosuppressive macrophage infiltration into the GBM microenvironment, thus exacerbating GBM oncogenesis. This research offers a comprehensive examination of DNAJC1’s influence on immune cell infiltration, particularly its interaction with immunosuppressive cell types, and delves into the molecular mechanisms underpinning these processes in GBM, proposing novel diagnostic and therapeutic strategies.

## Materials and methods

### Datasets

We sourced a comprehensive dataset comprising 10,534 tumor samples across various subtypes, including matched normal controls, from The Cancer Genome Atlas (TCGA) to analyze DNAJC1 expression patterns in pan-cancer contexts. This dataset encompassed RNA-seq data from 689 GBM patients and 1157 normal controls. Additional data for primary GBM specimens were obtained from the China Glioma Genome Atlas (CGGA), consisting of 156 cases and 5 controls, and the Gene Expression Omnibus (GEO), which included 52 GBM specimens and 20 controls from the GSE29796 series. These datasets facilitated a detailed investigation into the differential expression of DNAJC1 in GBM and its potential association with clinical parameters, including WHO grade, IDH status, 1p/19q co-deletion, and histological subtypes.

### Bioinformatics analysis

The TCGA GBM dataset was bifurcated into high and low DNAJC1 expression groups. We sequenced the transcriptomes of these cohorts to pinpoint differentially expressed genes (DEGs) and their enriched functions or pathways. The R software package (Version 4.2.1) was used for group classification and DEG calculation. Gene Ontology (GO) and Kyoto Encyclopedia of Genes and Genomes (KEGG) analyses identified enriched cellular components (CC), biological processes (BP), molecular functions (MF), and signaling pathways. Gene Set Enrichment Analysis (GSEA) corroborated these findings by assessing biological functions and pathway enrichments, adhering to the cutoff criteria of *P* < 0.05, |NES| > 1, and FDR < 0.25. We extracted gene markers for 24 immune cell types from previous research to analyze immune infiltration, employing single-sample GSEA (ssGSEA) with the GSVA R package using TCGA-COADREAD datasets (Bindea et al. 2013). Spearman correlation tests quantified the association between DNAJC1 expression and immune cell infiltration. Visualization was achieved using the ggplot2 R package.

### Immunohistochemistry on human glioma specimens

Glioma tissues, including a variety of histological grades and normal brain tissues, were procured from the Pathology Department of Xijing Hospital, Air Force Medical University, Xi’an, China. The collection encompassed tissue chips from 63 patients, treated from 2010 to 2015. Ethical approval was granted by the institution’s ethics committee, and informed consent was obtained from all participants. Tissue preparation entailed meticulous microdissection.

For immunohistochemistry (IHC), sections underwent deparaffinization, rehydration through graded ethanol, and antigen retrieval by boiling. Blocking of endogenous peroxidase was with 3% H_2_O_2_ in methanol. Primary antibodies were incubated overnight at 4 °C in a humidified chamber. Subsequent incubation with secondary antibodies and streptavidin-conjugated horseradish peroxidase was conducted. Visualization utilized 3,3′-diaminobenzidine (DAB), and hematoxylin counterstaining followed. Sections were dehydrated and mounted in Eukitt medium.

### Cell culture

GBM cell lines U251 and U87, and the mononuclear cell line THP-1, were sourced from the Cell Bank of the Chinese Academy of Sciences. Cultivation occurred in RPMI-1640 medium, enriched with 10% fetal bovine serum and 1% penicillin–streptomycin, in a humidified 37 °C incubator with 5% CO_2_.

### Plasmid construction and lentivirus production

Plasmids containing specific shRNA sequences targeting DNAJC1 were constructed as described: shDNAJC1_1: 5′ GCAGCTCAACTTCTACCAGTT 3′; shDNAJC1_2: 5′ GGGTCATTATGCTGTGGTTTG 3′; shDNAJC1_3: 5′ AGGTACAAGTTGCTGGTTGAA 3′. A random control sequence for targeting was also generated: 5′ ACTACCGTTGTTATAGGTG 3′. The DNAJC1 shRNAs and control shRNA were synthesized and inserted downstream of a U6 promoter in the pLVX-U6-EF1α-GFP-Puro lentiviral vector. Validation of all cloning steps was conducted through sequencing. The transfer plasmid containing the shRNA, the viral envelope plasmid, and the packaging plasmid were co-transfected into HEK293T cells using PEI transfection reagent (Life-iLab Biotech Inc., Shanghai, China). After 48 and 72 h post-transfection, lentivirus-containing supernatants were harvested, filtered through a 0.45 µm filter (Millipore, MA, USA), and subsequently combined with PEG6000 overnight at 4 °C. The mixture was concentrated via centrifugation at 4500 × g for 30 min at 4 °C. U251 and U87 cells were transduced with the lentivirus at a multiplicity of infection (MOI) of 50 accompanied by 10 µg/mL polybrene. Following 72 h of viral infection, the culture medium was supplemented with 2 μg/mL puromycin (Beyotime, Shanghai, China), and the cells were maintained for an additional week to establish stable cell lines expressing both GFP and shRNA.

### Protein isolation and western blot analysis

Total protein was isolated from cell lysates using RIPA buffer (Sangon, Shanghai, China) with a protease inhibitor cocktail (Genstar, Shenzhen, China). Protein concentration was determined via bicinchoninic acid assay (BCA kit, Biosharp, Hefei, China). Then, 25 μg of protein per sample was resolved on a 10% SDS-PAGE gel at 110 V and transferred to a PVDF membrane (Millipore at 300 mA). Blocking was performed using 5% BSA in TBST for 1 h at room temperature. Primary antibodies targeting DNAJC1 (1:2000), E-cadherin (1:5000), Vimentin (1:2000) (all from Proteintech, Wuhan, China), β-actin (1:1000), and GAPDH (1:1000) (both from Cell Signaling Technology, MA, USA) were applied and incubated overnight at 4 °C. Following washes with TBST, membranes were incubated with secondary antibodies for 1 h at room temperature. Protein bands were visualized using a FluorChem FC2 system (Alpha Innotech, CA, USA) as per the manufacturer’s protocol.

### Growth curve assay

Cell proliferation was assessed using a CCK-8 kit (Beyotime, Shanghai, China). Cells (2 × 10^3^ per well) were seeded in 96-well plates with 200 μL of 10% FBS medium. Post attachment, 10 μL of CCK-8 solution was added and incubated at 37 °C for 90 min. Absorbance at 450 nm was measured with a microplate reader (Bio-Rad, CA, USA). Assays were conducted at 0, 24, 48, and 72 h post-seeding and replicated thrice.

### Colony formation assay

Logarithmically growing cells (2 × 10^3^) were plated in a 6-cm dish with 5 mL of 10% FBS medium, ensuring even dispersion. After 2–3 weeks of incubation at 37 °C and 5% CO_2_, cells were fixed with methanol, stained with Giemsa, and colonies were counted upon dish inversion.

### Cell apoptosis and cycle analyses

Flow cytometry was employed for apoptosis and cell cycle analyses. For apoptosis, cells (5 × 10^5^) were cultured for 24, 48, or 72 h, then exposed to 2% FBS medium for 24 h. Post-harvesting, cells were stained with 7-AAD and Annexin V-FITC (BD-Biosciences, NJ, USA) for 30 min at 4 °C in darkness. Analysis was performed using an EPICS XL flow cytometer (Beckman Coulter, CA, USA) with CellQuest software. For cell cycle analysis, cells were fixed in 70% ethanol overnight at 4 °C, rinsed, and stained with PI and RNase (BD-Biosciences, NJ, USA). A FACScan flow cytometer was used for detection. Each assay was conducted in triplicate.

### Wound healing and transwell assay

Cell migration was evaluated through wound healing and transwell assays. For wound healing, cells (5 × 10^5^) in a 6-well plate were grown to 90% confluence, scratched with a pipette tip, and imaged at 0 and 24 h. In transwell assays, cells (1 × 10^4^) were placed in the upper chamber of a transwell setup with complete medium as chemoattractant below. After 48 h, cells were fixed, stained with crystal violet, and counted under a microscope. Each assay was performed thrice.

### Cell coculture

A cell coculture system was established by differentiating THP-1 cells into macrophages using 100 ng/mL phorbol 12-myristate 13-acetate (PMA, Sigma, MO, USA). After 24 h, the macrophages were polarized into M2 phenotype by incubating with 20 ng/mL IL-4 for an additional 48 h. For the coculture, 1 × 10^5^ macrophages were suspended in 500 μL of serum-free medium and seeded onto the upper transwell chamber membrane without Matrigel coating (24-well, 8 μm; Millipore). The lower compartment was filled with 500 μL of GBM cell supernatants. Following a 24-h incubation period, the transwell chamber was washed thrice with PBS, stained with 0.1% crystal violet for 20 min, air-dried, photographed, and cell counts were conducted in at least five randomly chosen fields. Additionally, 1 × 10^6^ THP-1 cells were induced by PMA and differentiated into macrophages. Then, 1 × 10^6^ U251 tumor cells or DNAJC1-silenced U251 tumor cells were co-cultured with THP-1 for 48 h. The expression of CD163 was assessed using flow cytometry.

### Statistical analysis

Data were analyzed with SPSS 25.0, presented as mean ± SD, and derived from a minimum of three assays. R software (Version 4.2.1) was employed for bioinformatics analysis. Variations between two groups were assessed using a two-tailed Student’s *t* test, while one-way ANOVA with Bonferroni’s post hoc test compared multiple groups. Survival analysis was conducted using Kaplan–Meier and log-rank tests, correlations were examined through Spearman’s test, and group characteristics were analyzed using Cox regression. Diagnostic accuracy was evaluated using ROC curve and AUC analyses. Significance levels were indicated as follows: *, *P* < 0.05; **, *P* < 0.01; ***, *P* < 0.001.

## Results

### DNAJC1 expression is elevated and may play a role in human GBM tumorigenesis

**Fig. 1:**
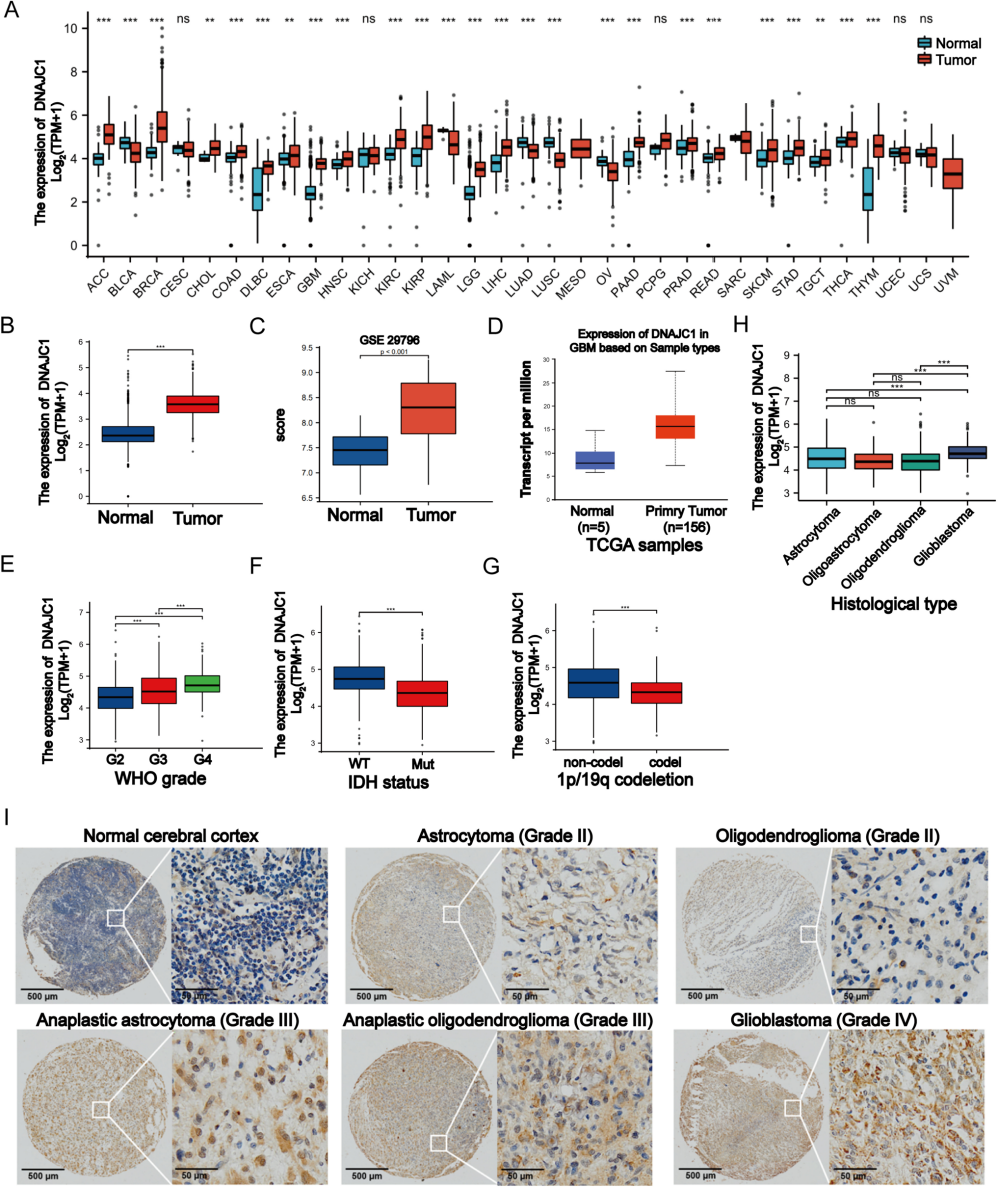
Correlation between DNAJC1 expression and clinicopathological characteristics of GBM. **A** DNAJC1 expression in different cancers from the TCGA database. ACC, adrenocortical carcinoma; BLCA, bladder urothelial carcinoma; BRCA, breast invasive carcinoma; CESC, cervical squamous cell carcinoma and endocervical adenocarcinoma; CHOL, cholangiocarcinoma; COAD, colon adenocarcinoma; DLBC, lymphoid neoplasm diffuse large B-cell lymphoma; ESCA, esophageal carcinoma; GBM, glioblastoma multiforme; HNSC, head and neck squamous cell carcinoma; KICH, kidney chromophobe; KIRC, kidney renal clear cell carcinoma; KIRP, kidney renal papillary cell carcinoma; LAML, acute myeloid leukemia; LGG, low grade glioma; LIHC, liver hepatocellular carcinoma; LUAD, lung adenocarcinoma; LUSC, lung squamous cell carcinoma; MESO, mesothelioma; OV, ovarian serous cystadenocarcinoma; PAAD, pancreatic adenocarcinoma; PCPG, pheochromocytoma and paraganglioma; PRAD, prostate adenocarcinoma; READ, rectum adenocarcinoma; SARC, sarcoma; SKCM, skin cutaneous melanoma; STAD, stomach adenocarcinoma; TGCT, testicular germ cell tumors; THCA, thyroid carcinoma; THYM, thymoma; UCEC, uterine corpus endometrial carcinoma; UCS, uterine carcinosarcoma; UVM, uveal melanoma. The expression of DNAJC1 in GBM samples and normal tissues from **B** TCGA, **C** GSE29796 or **D** CGGA. The expression of DNAJC1 in different **E** glioma grades, **F** IDH mutant status, **G** chromosome 1p/19q codeletion or **H** histological types. **I** Representative immunohistochemical staining of DNAJC1 in different histological gliomas using a tissue chip. * *P* < 0.05; ** *P* < 0.01; *** *P* < 0.001

To examine the expression of DNAJC1 in tumors, we analyzed data from TCGA database. Our analysis revealed a significant upregulation of DNAJC1 in 20 different tumor types, including GBM and brain lower-grade glioma (LGG), compared to adjacent normal tissues (Fig. 1A). To validate these findings, we also analyzed RNA-seq data from TCGA (Fig. 1B), GSE29796 (Fig. 1C), and CGGA databases (Fig. 1D). Consistently, the expression of DNAJC1 was significantly higher in GBM specimens compared to normal specimens, indicating a positive association between DNAJC1 and GBM.

Furthermore, we investigated the correlation between DNAJC1 and various clinical factors using the TCGA database (Table 1). Our results demonstrated that the expression of DNAJC1 gradually increased with the progression of GBM from WHO grade I–IV (Fig. 1E). Additionally, GBM specimens with IDH mutations exhibited lower levels of DNAJC1 compared to those with wild-type IDH (Fig. 1F). Moreover, DNAJC1 expression was significantly lower in GBM specimens with chromosome 1p/19q co-deletion compared to those without co-deletion (Fig. 1G). Notably, GBM showed higher levels of DNAJC1 expression compared to less malignant subtypes such as astrocytoma, oligoastrocytoma, and oligodendroglioma (Fig. 1H).

**Table 1:** Association between DNAJC1 expression and clinical characteristics of glioma patients

Characteristic	Low expression of DNAJC1	High expression of DNAJC1	*P*
*n*	348	348	
WHO grade, *n* (%)			<0.001
G2	150 (23.6%)	74 (11.7%)	
G3	114 (18%)	129 (20.3%)	
G4	43 (6.8%)	125 (19.7%)	
IDH status, *n* (%)			<0.001
WT	69 (10.1%)	177 (25.8%)	
Mut	275 (40.1%)	165 (24.1%)	
1p/19q codeletion, *n* (%)			<0.001
Codel	115 (16.7%)	56 (8.1%)	
Non-codel	231 (33.5%)	287 (41.7%)	
Primary therapy outcome, *n* (%)			0.218
PD	55 (11.9%)	57 (12.3%)	
SD	90 (19.5%)	57 (12.3%)	
PR	38 (8.2%)	26 (5.6%)	
CR	83 (18%)	56 (12.1%)	
Gender, *n* (%)			0.592
Female	145 (20.8%)	153 (22%)	
Male	203 (29.2%)	195 (28%)	
Race, *n* (%)			0.692
Asian	5 (0.7%)	8 (1.2%)	
Black or African American	17 (2.5%)	16 (2.3%)	
White	320 (46.9%)	317 (46.4%)	
Age, *n* (%)			<0.001
≤60	297 (42.7%)	256 (36.8%)	
>60	51 (7.3%)	92 (13.2%)	
Histological type, *n* (%)			<0.001
Astrocytoma	102 (14.7%)	93 (13.4%)	
Glioblastoma	43 (6.2%)	125 (18%)	
Oligoastrocytoma	83 (11.9%)	51 (7.3%)	
Oligodendroglioma	120 (17.2%)	79 (11.4%)	
OS event, *n* (%)			<0.001
Alive	252 (36.2%)	172 (24.7%)	
Dead	96 (13.8%)	176 (25.3%)	
DSS event, *n* (%)			<0.001
Alive	257 (38.1%)	174 (25.8%)	
Dead	85 (12.6%)	159 (23.6%)	
PFI event, *n* (%)			<0.001
Alive	203 (29.2%)	147 (21.1%)	
Dead	145 (20.8%)	201 (28.9%)	
Age, median (IQR)	41 (33, 54)	51 (36, 61.25)	<0.001

To further evaluate the expression pattern of DNAJC1 in different glioma grades, we performed immunohistochemical staining on tissue samples obtained from patients with various tumor grades as well as normal brain tissue samples. Our results demonstrated a gradual increase in DNAJC1 expression from normal cerebral cortex to astrocytoma (WHO grade II) or oligoastrocytoma (WHO grade II), and further to anaplastic astrocytoma (WHO grade III) and anaplastic oligodendroglioma (WHO grade III). Notably, the highest expression level of DNAJC1 was observed in GBM (WHO grade IV) (Fig. 1I). These findings suggest that DNAJC1 is positively associated with clinical progression and may potentially act as a tumor activator in human GBM.

### DNAJC1 serves as an unfavorable prognostic *indicator* and a potential diagnostic biomarker for human GBM

**Fig. 2:**
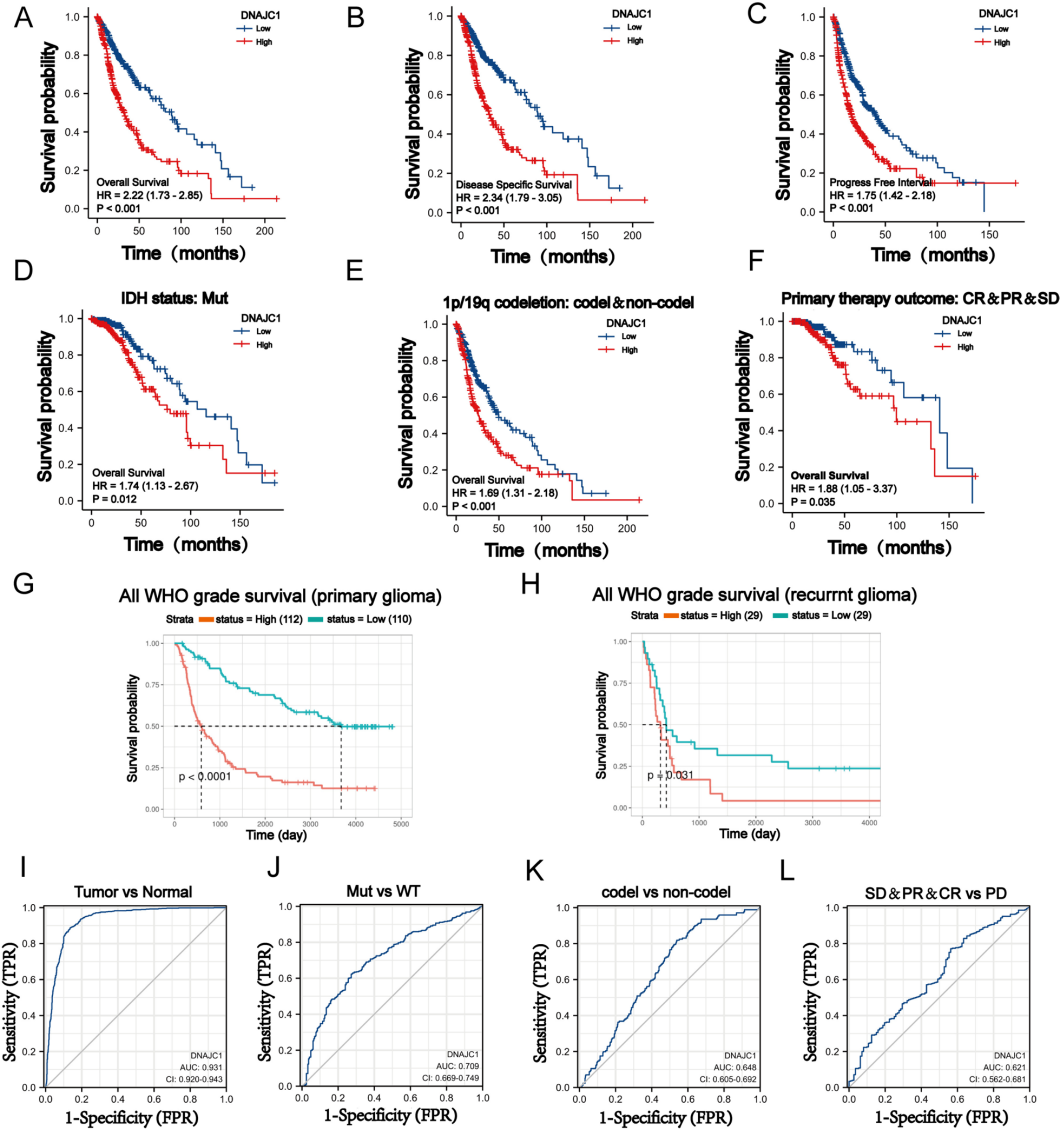
Correlation between DNAJC1 expression and GBM survival prognosis. Correlation between DNAJC1 expression and **A** overall survival, **B** disease-specific survival or **C** progression-free interval in GBM patients. Correlation between DNAJC1 expression and the overall survival of **D** IDH mutation status, **E** chromosome 1p/19q noncodeletion or **F** primary therapy outcome in GBM patients. Correlation between DNAJC1 expression and the overall survival of GBM patients with **G** primary tumors or **H** recurrent tumors. Receiver operating characteristic (ROC) curve analyses were used to distinguish DNAJC1 expression in **I** primary GBM tumors, **J** IDH mutation, **K** 1p/19q codeletion or **L** primary therapy outcome in GBM patients

To evaluate the relationship between DNAJC1 expression and patient survival, we analyzed survival data obtained from the TCGA database. GBM patients were divided into two cohorts based on their DNAJC1 expression: high and low. The data, illustrated in Fig. 2A–C, showed that patients with elevated DNAJC1 expression have significantly reduced overall survival (OS), disease-specific survival (DSS), and progression-free interval (PFI) in contrast to those with diminished expression. In scenarios involving IDH mutation, 1p/19q codeletion, or primary therapy outcome, the high-expression cohort consistently presented with poorer OS outcomes (Fig. 2D–F). Both univariate and multivariate analyses corroborated the status of DNAJC1 expression as an independent prognostic factor for GBM (Table 2). The CGGA database analysis corroborated these findings, revealing an inverse relationship between DNAJC1 expression and OS in patients with either initial or recurrent GBM (Fig. 2G, H), further supporting DNAJC1’s role as a negative prognostic indicator.

**Table 2:** Univariate and multivariate analyses of factors associated with overall survival

Characteristics	Total (*N*)	Univariate analysis		Multivariate analysis	
		Hazard ratio (95% CI)	*P* value	Hazard ratio (95% CI)	*P* value
Histological type	669				
Glioblastoma	160	Reference			
Astrocytoma	192	0.151 (0.109–0.209)	<0.001	0.144 (0.044–0.473)	0.001
Oligoastrocytoma	128	0.093 (0.060–0.144)	<0.001	0.160 (0.047–0.543)	0.003
Oligodendroglioma	189	0.085 (0.058–0.124)	<0.001	0.107 (0.032–0.360)	<0.001
Age	669				
≤60	530	Reference			
>60	139	4.716 (3.609–6.161)	<0.001	4.004 (2.370–6.763)	<0.001
Primary therapy outcome	443				
PD	103	Reference			
SD	144	0.381 (0.250–0.580)	<0.001	0.373 (0.222–0.628)	<0.001
PR	62	0.138 (0.055–0.342)	<0.001	0.191 (0.067–0.541)	0.002
CR	134	0.131 (0.063–0.274)	<0.001	0.175 (0.081–0.380)	<0.001
IDH status	660				
WT	237	Reference			
Mut	423	0.102 (0.077–0.135)	<0.001	0.404 (0.228–0.717)	0.002
1p/19q codeletion	663				
Codel	167	Reference			
Non-codel	496	4.635 (2.963–7.251)	<0.001	1.122 (0.567–2.220)	0.740
WHO grade	612				
G2	215	Reference			
G3	237	3.102 (2.030–4.739)	<0.001	1.911 (1.161–3.147)	0.011
G4	160	19.164 (12.573–29.209)	<0.001		
DNAJC1	669				
Low	334	Reference			
High	335	2.295 (1.771–2.974)	<0.001	1.064 (0.667–1.698)	0.795

In addition, receiver operating characteristic (ROC) curve analysis highlighted DNAJC1’s capacity to differentiate between primary GBM tumors and normal tissue controls, with an impressive area under the curve (AUC) of 0.931 (Fig. 2I). The discriminative power of DNAJC1 was also consistent across variables such as IDH mutation status, 1p/19q codeletion status, and primary therapy outcomes, yielding AUC values of 0.709, 0.648, and 0.621, respectively (Fig. 2J–L). These findings suggest that DNAJC1 is a promising diagnostic biomarker for GBM in a clinical context.

### DNAJC1 enhances proliferation and suppresses apoptosis in GBM cells in vitro

**Fig. 3:**
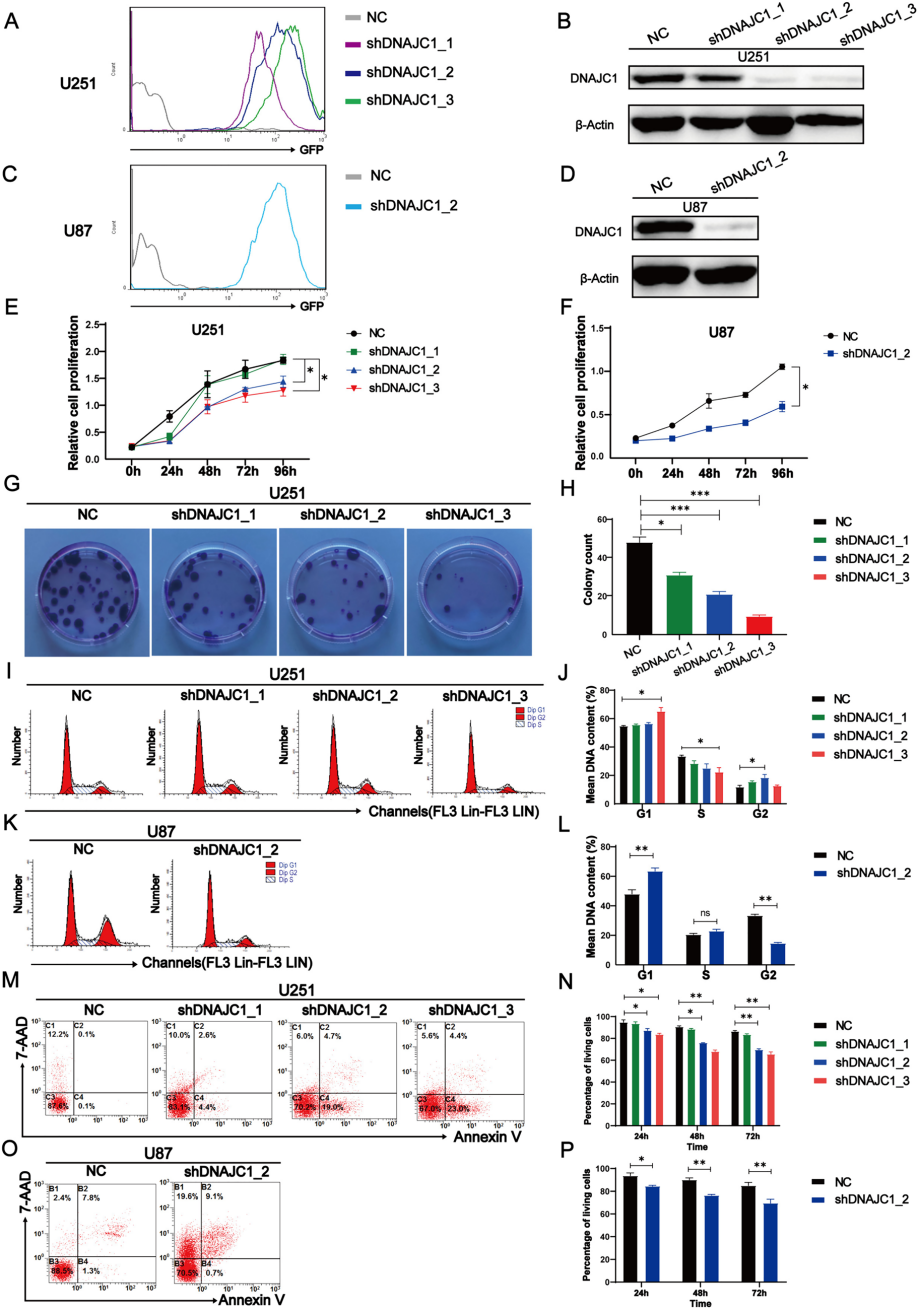
DNAJC1 silencing inhibits proliferation and promotes apoptosis in GBM cells. **A**–**D** Establishment of GMB cell lines stably expressing control shRNA or DNAJC1-targeting shRNA. U251 and U87 cells were transduced with lentivirus encoding GFP and shRNA. Puromycin (2 μg/mL) was added to the culture medium 72 h post-virus infection, and the cells were cultured for another week to obtain stable GFP- and shRNA-expressing cell lines. Flow cytometry analysis of GFP-positive **A** U251 cells and **C** U87 cells was performed. Western blot analysis was used to detect DNAJC1 expression in DNAJC1- silencing **B** U251 cells and **D** U87 cells. **E**–**H** DNAJC1 silencing inhibits GBM cell growth. A growth curve assay was performed with DNAJC1- silencing **E** U251 cells and **F** U87 cells. **G** Colony formation assay and **H** statistical analysis were performed with DNAJC1- silencing U251 cells. Cell cycle experiments and statistical analyses were performed in DNAJC1- silencing **I**,**J** U251 cells and **K**,**L** U87 cells. **M**–**P** U251 or U87 cells were cultured in medium containing 2% FBS for 24 h, 48 h, or 72 h before harvesting. Flow cytometry analysis was performed with Annexin V-FITC and 7AAD staining to detect apoptosis at 72 h (**M**, **O**) or over a period of time (**N**, **P**) after serum deprivation

To elucidate DNAJC1’s oncogenic function in GBM, we performed in vitro experiments using GBM cell lines. U251 cells were infected with lentiviruses carrying GFP alongside various DNAJC1-targeting shRNAs. GFP-positive cells underwent selection via puromycin (Fig. 3A). Western blot analysis revealed marked downregulation of DNAJC1 in cells treated with shDNAJC1_2 and shDNAJC1_3, and a modest reduction with shDNAJC1_1, relative to controls (Fig. 3B). For validation, we generated U87 GBM cell lines transfected with either a control vector or shDNAJC1_2. Consistent expression patterns were confirmed in both U87 and U251 cells through flow cytometry and western blotting (Fig. 3C, D).

Proliferation assays indicated that both U251 and U87 cells with DNAJC1 knockdown (shDNAJC1_2 and shDNAJC1_3) exhibited reduced growth compared to control vector-transfected cells (Fig. 3E, F). Colony formation assays showed a substantial reduction in colony numbers in DNAJC1-silenced U251 cells (Fig. 3G, H). Cell cycle analysis indicated a decrease in S-phase fraction and an increase in G0/G1 and G2 phases upon DNAJC1 silencing in both cell lines (Fig. 3I–L). Furthermore, apoptotic rates increased in a time-dependent manner following DNAJC1 downregulation in serum-deprived U251 (Fig. 3M, N) and U87 (Fig. 3O, P) cells, as determined by flow cytometry with Annexin V-FITC and 7-AAD staining. Collectively, these data imply that DNAJC1 facilitates GBM cell proliferation, expedites cell cycle progression, and inhibits apoptosis in vitro.

### DNAJC1 influences ECM organization and immune response in the GBM microenvironment

**Fig. 4:**
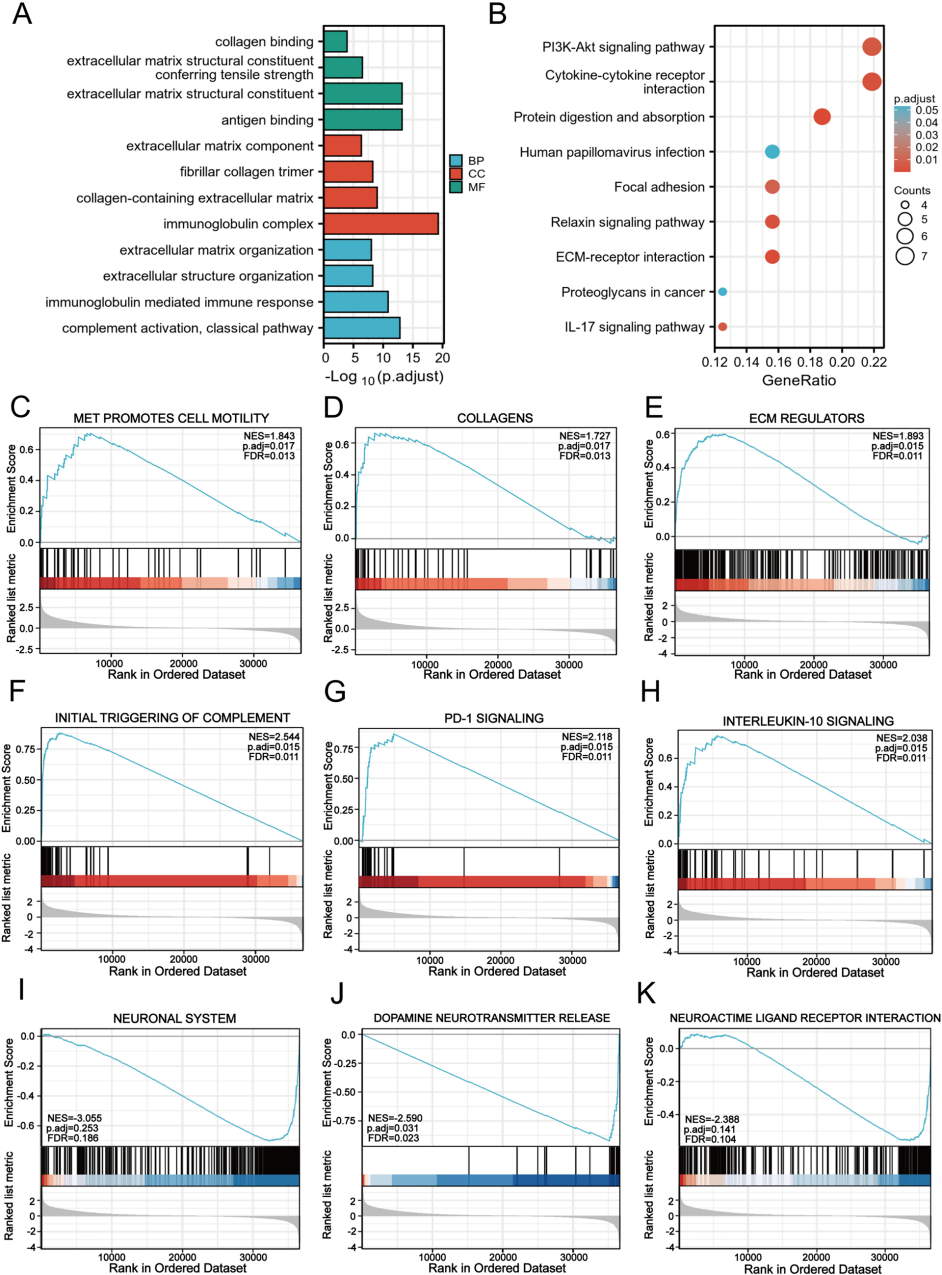
Functional enrichment analysis of DNAJC1 in GBM from the TCGA database. **A** Enriched Gene Ontology terms of DNAJC1 coexpression genes in GBM based on biological processes, cellular components, and molecular function. **B** KEGG analysis of GBM patients with high DNAJC1 expression. Enrichment plots from GSEA, including **C** MET promotes the cell motility pathway, **D** collagens pathway, **E** ECM regulators pathway, **F** initial triggering of the complement pathway, **G** PD-1 signaling pathway, **H** interleukin 10 signaling pathway, **I** neuronal system pathway, **J** dopamine neurotransmitter release pathway, and **K** neuroactive ligand receptor interaction pathway

To decipher DNAJC1’s role in the evolution and advancement of GBM, we conducted Gene Ontology (GO) and Kyoto Encyclopedia of Genes and Genomes (KEGG) pathway analyses on GBM samples exhibiting elevated DNAJC1 expression. The analysis presented in Fig. 4A identified significant enrichment in biological functions related to the extracellular matrix structural constituent (GO-MF), the immunoglobulin complex (GO-CC), and complement activation (GO-BP). The KEGG pathway analysis revealed a notable correlation of the DNAJC1-associated gene cluster with pivotal processes and pathways, including the PI3K/AKT signaling pathway, cytokine-cytokine receptor interaction, focal adhesion, ECM-receptor interaction, and the IL-17 signaling pathway (Fig. 4B). These data point to DNAJC1’s possible involvement in modulating the ECM and immune response within the GBM microenvironment.

Gene set enrichment analysis (GSEA) further corroborated these observations in the high DNAJC1 expression sample subset. We focused on gene clusters meeting the threshold of a nominal *P* value (NOM) <0.05 and a false discovery rate (FDR) *q* < 0.25, calculating the normalized enrichment score (NES) to ascertain specific pathway enrichments. The analysis revealed a significant enrichment in genes related to the promotion of cell motility by MET, collagen synthesis, and regulation of extracellular matrix components, reinforcing the critical role of DNAJC1 in modulating the extracellular matrix (Fig. 4C–E). Moreover, the enriched gene cluster was associated with pathways involved in the initial triggering of complement activation, shedding light on the biological event or signaling process that initiates complement protein activation and triggers a cascade reaction (Fig. 4F). Furthermore, PD-1 signaling (Fig. 4G) and interleukin 10 signaling (Fig. 4H) were also enriched, highlighting DNAJC1’s close association with immune response mechanisms within the GBM microenvironment. Importantly, DNAJC1 was identified as a negative regulator of neuronal system development and exhibited enrichment in related pathways such as the neuronal system (Fig. 4I), dopamine neurotransmitter release (Fig. 4J), and neuroactive ligand-receptor interaction (Fig. 4K). In summary, the findings underscore DNAJC1’s central role in coordinating ECM organization and immune dynamics, creating an environment conducive to GBM progression, invasion, and immune evasion.

### DNAJC1 knockdown inhibits GBM cell migration and EMT

**Fig. 5:**
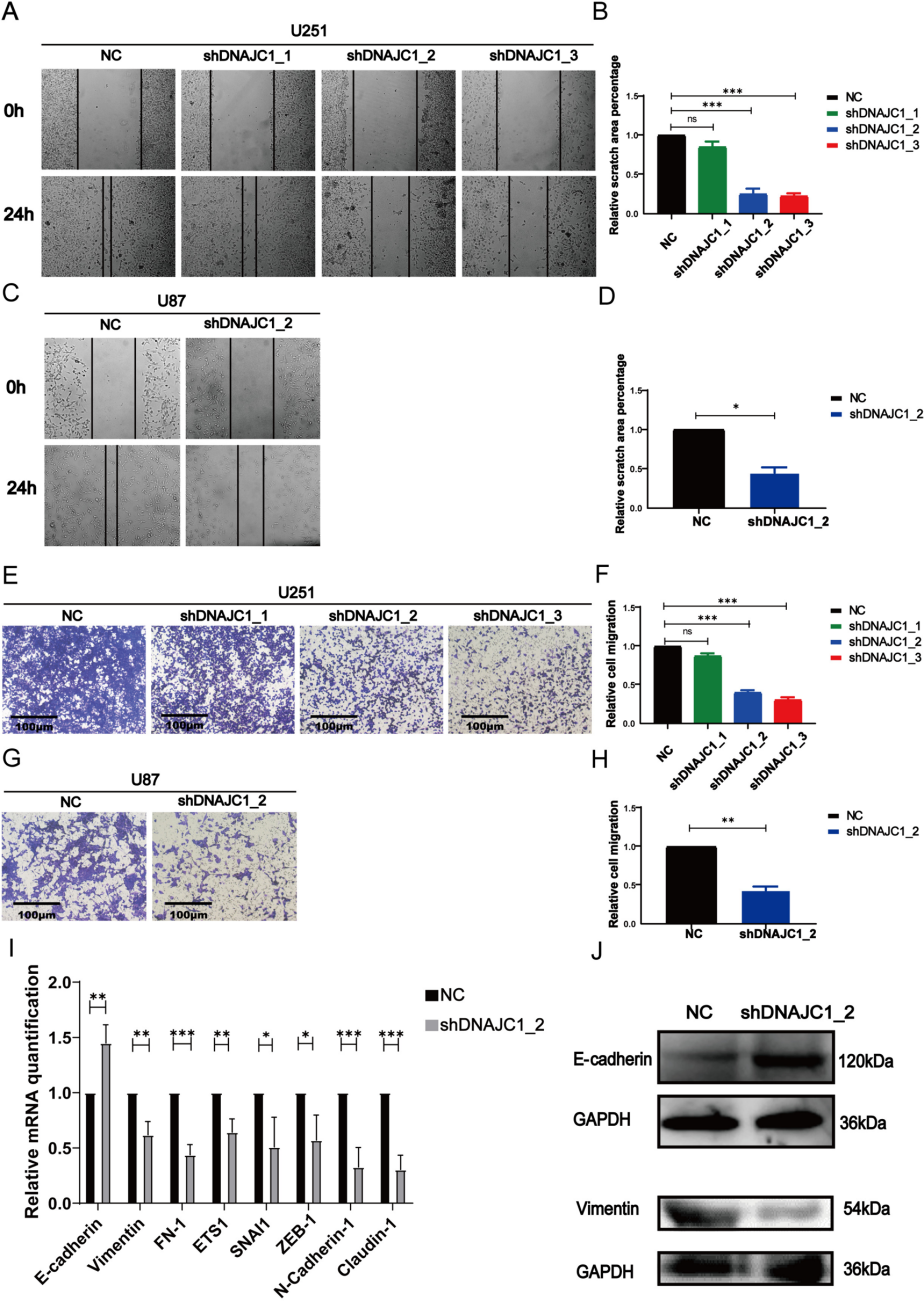
DNAJC1 promotes the migration of GBM cells and induces the EMT process. Wound healing experiments were conducted along with statistical analysis to assess the migratory capacity of DNAJC1- silencing **A**,**B** U251 cells and **C**,**D** U87 cells. The migration ability of **E**,**F** U251 cells and **G**,**H** U87 cells was examined using the transwell assay. **I** qRT-PCR analysis was performed to assess the expression of EMT markers in U251 cells with DNAJC1 knocked down and control cells. **J** Western blot analysis was conducted to examine the expression of the epithelial marker E-cadherin and the mesenchymal marker Vimentin in U251 cells with DNAJC1 knocked down and control cells

Reflecting on the GO and GSEA results that linked DNAJC1 expression to ECM organization, we explored the gene’s effects on GBM cell motility. Wound healing assays demonstrated a marked reduction in migratory capabilities of U251 and U87 cells with silenced DNAJC1 (Fig. 5A–D). Similarly, transwell assays indicated fewer migrating cells on the membranes of the lower chambers when DNAJC1 expression was inhibited in U251 and U87 cells (Fig. 5E–H). These results robustly suggest that DNAJC1 is instrumental in promoting GBM cell migration in vitro.

Epithelial-mesenchymal transition (EMT) plays a pivotal role in the metastasis of various solid tumors. We assessed the role of DNAJC1 in EMT using quantitative real-time PCR (qRT-PCR) to measure the impact of DNAJC1 knockdown on EMT marker expression in U251 cells. A substantial decrease in the mRNA expression of mesenchymal markers, such as Vimentin, FN1, ETS1, SNAI1, and ZEB1, was observed upon suppression of DNAJC1. Conversely, the epithelial marker E-cadherin showed increased expression when DNAJC1 was inhibited (Fig. 5I). This observation was further supported by Western blot analysis, which revealed an increase in E-cadherin levels and a decrease in Vimentin levels in U251 cells with reduced DNAJC1 expression (Fig. 5J). These results confirm the involvement of DNAJC1 in promoting epithelial-mesenchymal transition (EMT), thereby influencing the migratory and invasive characteristics of GBM cells.

### DNAJC1 augments immunosuppressive cell infiltration in the GBM microenvironment

**Fig. 6:**
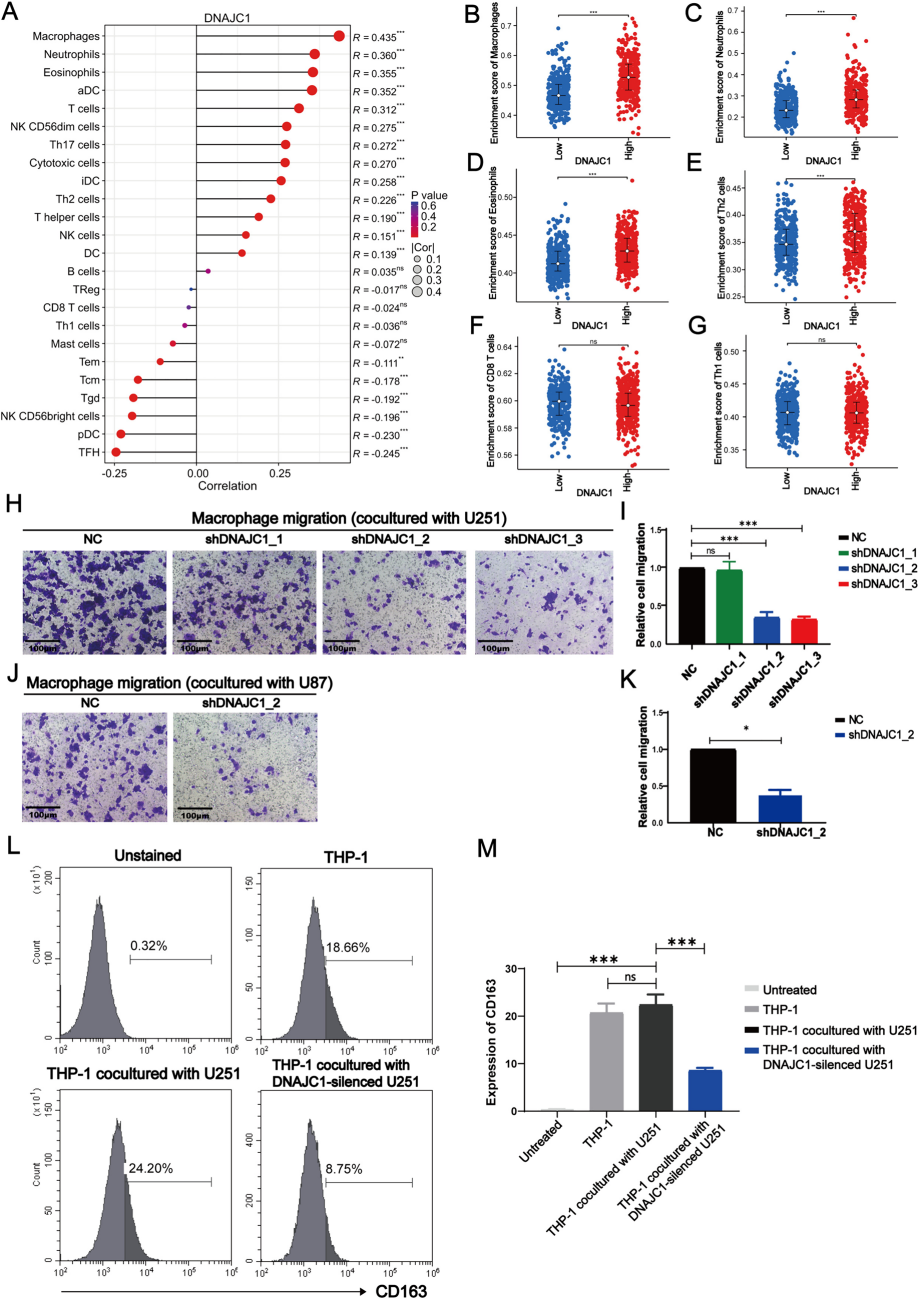
Correlation analysis between DNAJC1 expression and macrophage infiltration in GBM. **A** Correlation between DNAJC1 expression and 24 immune cells. Relationship between DNAJC1 expression level and GBM infiltrating **B** macrophages, **C** neutrophils, **D** Eosinophils, **E** Th2 cells, **F** CD8^+^ T cells, and **G** Th1 cells. **H**–**K** Coculture and recruitment experiments were performed using GBM cells and macrophages. Macrophages, differentiated from THP-1 cells by PMA, were seeded in the upper transwell chambers, while DNAJC1- silencing **H**,**I** U251 cells or **J**,**K** U87 cells were placed in the lower transwell chambers. Following 24 h of incubation, macrophages that migrated through the chamber membrane were stained with 0.1% crystal violet and counted for statistical analysis. **L**,**M** Flow cytometry was employed to analyze the expression of the M2 marker CD163 on macrophages after coculture with DNAJC1-silenced U251 cells or control cells

Our investigation delved into the role of DNAJC1 in modulating immune cell infiltration within GBM (Fig. 6A; Table 3). A distinct link was established between DNAJC1 expression and the presence of immunosuppressive cell types, such as macrophages, neutrophils, and T helper type 2 (Th2) cells, known to facilitate GBM tumorigenesis within the tumor microenvironment (TME) Notably, groups with high DNAJC1 expression had significantly greater enrichment of macrophages (Fig. 6B), neutrophils (Fig. 6C), Eosinophils (Fig. 6D), and Th2 cells (Fig. 6E) when compared to low-expression groups. These results point to DNAJC1’s involvement in the recruitment of pro-tumorigenic immune cells within the GBM microenvironment. In contrast, there was no discernible association between DNAJC1 expression and the infiltration of anti-tumorigenic immune cells, such as CD8^+^ T cells and Th1 cells, which were similarly distributed across both high-expression and low-expression DNAJC1 groups (Fig. 6F, G). Thus, our data underscore DNAJC1’s role in fostering the infiltration of immune cells that support GBM tumorigenesis.

**Table 3:** Correlation between the expression of DNAJC1 and immune cell infiltration

Molecular	Immune cell	Pearson	Pearson	p-Pearson	Spearman	Spearman	p-Spearman
DNAJC1	aDC	9.7384	0.3519	4.73e−21	3.071e+07	0.3956	0
DNAJC1	B cells	0.91858	0.0354391	0.3586	4.923e+07	0.0310388	0.4214
DNAJC1	CD8 T cells	−0.61375	−0.0236869	0.5396	5.312e+07	−0.0456498	0.2369
DNAJC1	Cytotoxic cells	7.26572	0.270068	1.03e−12	3.63e+07	0.285567	5.52e−14
DNAJC1	DC	3.62581	0.138621	0.0003	4.495e+07	0.115231	0.0028
DNAJC1	Eosinophils	9.828	0.354732	2.18e−21	3.177e+07	0.374668	0
DNAJC1	iDC	6.9154	0.257933	1.09e−11	3.531e+07	0.304885	8.09e−16
DNAJC1	Macrophages	12.5078	0.434821	2.05e−32	2.64e+07	0.480309	0
DNAJC1	Mast cells	−1.86909	−0.0719684	0.0620	5.519e+07	−0.0862965	0.0252
DNAJC1	Neutrophils	10.0009	0.36017	4.82e−22	3.067e+07	0.396271	0
DNAJC1	NK CD56bright cells	−5.18025	−0.196098	2.93e−07	6.077e+07	−0.196096	3.1e−07
DNAJC1	NK CD56dim cells	7.41073	0.275053	3.79e−13	3.648e+07	0.281931	1.18e−13
DNAJC1	NK cells	3.94386	0.150516	8.86e−05	4.237e+07	0.165973	1.55e−05
DNAJC1	pDC	−6.12936	−0.230263	1.5e−09	6.238e+07	−0.227951	2.21e−09
DNAJC1	T cells	8.51574	0.312303	1.08e−16	3.475e+07	0.316087	5.69e−17
DNAJC1	T helper cells	5.00315	0.18964	7.21e−07	4.17e+07	0.17912	3.04e−06
DNAJC1	Tcm	−4.69668	−0.178404	3.21e−06	6.065e+07	−0.193783	4.29e−07
DNAJC1	Tem	−2.88104	−0.11054	0.0041	5.72e+07	−0.125924	0.0011
DNAJC1	TFH	−6.55085	−0.245174	1.14e−10	6.226e+07	−0.225591	3.61e−09
DNAJC1	Tgd	−5.0759	−0.192296	5e−07	5.542e+07	−0.0908845	0.0184
DNAJC1	Th1 cells	−0.924463	−0.0356658	0.3556	5.256e+07	−0.0346355	0.3696
DNAJC1	Th17 cells	7.30908	0.271561	7.67e−13	3.706e+07	0.270612	1.15e−12
DNAJC1	Th2 cells	6.02212	0.226443	2.84e−09	3.931e+07	0.226279	3.23e−09
DNAJC1	TReg	−0.428088	−0.0165239	0.6687	5.223e+07	−0.0281501	0.4660

To further investigate the interplay between DNAJC1 expression and macrophage infiltration, we employed a coculture system consisting of GBM cells and macrophages. When macrophages were cocultured with U251 and U87 cells with DNAJC1 knockdown (shDNAJC1_2 and shDNAJC1_3 for U251, shDNAJC1_2 for U87), we noted fewer labeled macrophages on the lower chamber membranes relative to control cells (Fig. 6H–K), aligning with our bioinformatic predictions (Fig. 6A, B). This suggests DNAJC1’s potential role in enhancing macrophage infiltration within the GBM microenvironment. Additionally, DNAJC1 suppression led to a decrease in the M2 macrophage marker CD163 (Fig. 6L, M), implying that GBM cell-derived DNAJC1 expression may be linked to the immunosuppressive M2 phenotype in macrophages.

## Discussion

The diagnostic paradigm for GBM has evolved to integrate molecular profiling alongside traditional histopathological examination. Clinically, molecular markers such as IDH mutations and 1p/19q codeletion are now routinely utilized for GBM diagnosis, prognosis assessment, and therapeutic decision-making (White et al. 2022). IDH, a critical enzyme in the Krebs cycle, catalyzes the conversion of isocitric acid into α-ketoglutarate (α-KG) and CO_2_, essential for energy production and biosynthetic precursor synthesis (Fan et al. 2021). Typically occurring in the early phases of glioma development, IDH mutations are prevalent in oligodendroglioma, astrocytoma, and secondary GBM, but rare in primary GBM (Han et al. 2020). Patients with IDH-mutant GBM generally have a protracted disease course and improved outcomes compared to those with wild-type IDH (Shi et al. 2022). The codeletion of the short arm of chromosome 1 and the long arm of chromosome 19 is often found in younger glioma patients (Hu et al. 2017). The WHO’s classification system identifies the 1p/19q codeletion as a distinctive marker for oligodendroglioma (Numan et al. 2022). As these chromosomal regions harbor essential genes for cellular growth and differentiation, this codeletion can inhibit GBM cell proliferation and vasculature formation, enhancing response to chemotherapy and ultimately leading to better patient prognoses (Wong et al. 2022).

Nonetheless, despite the employment of molecular markers such as IDH mutations and 1p/19q codeletion in the clinical management of GBM, patient outcomes remain dismal, with median survival rates of 14–17 months (Xie et al. 2020). Bioinformatic analysis of public databases has uncovered numerous novel differentially expressed genes that hold significant potential for improving GBM diagnostics and treatment strategies (Li et al. 2022; Wan et al. 2022; Manini et al. 2022).

This study commenced with an analysis of the TCGA database to ascertain the expression pattern of DNAJC1. The gene was significantly upregulated in at least 20 tumor subtypes, with notably high levels in GBM or LGG, suggesting a potential oncogenic role for DNAJC1. Further examination of TCGA, CGGA, and GEO databases corroborated the elevated expression of DNAJC1 in GBM tissues, which increased concomitantly with higher WHO tumor grades. Additionally, DNAJC1 expression was inversely correlated with both IDH mutation and 1p/19q codeletion. Kaplan–Meier survival analysis revealed that higher DNAJC1 expression was associated with poorer OS, DSS, and PFI in GBM patients. Conversely, patients with lower DNAJC1 expression displayed enhanced responses to chemotherapy, with higher complete response (CR), partial response (PR), and stable disease (SD) rates. These findings underscore DNAJC1’s potential as a prognostic biomarker and its diagnostic relevance in GBM.

The TME is instrumental in GBM growth and progression and consists of immune and non-immune elements that interact with tumor cells (White et al. 2022; Bikfalvi et al. 2023). Components such as collagen, laminin, and fibronectin are integral to the TME structure, alongside extracellular matrix elements, cancer-associated fibroblasts (CAFs), and immunosuppressive cytokines like IL-10 and IL-17 (Buoncervello et al. 2019; Li et al. 2019; Rivas et al. 2021). Notably, while traditionally considered protective, complement activation can also accelerate tumor progression through mediators such as C3a and C5a. These anaphylatoxins contribute to chronic inflammation, angiogenesis, and metastasis, presenting potential targets for cancer biomarkers and therapeutic interventions (Bouwens van der Vlis et al. 2018)(Meri et al. 2023). Our analyses via GO, KEGG, and GSEA identified DNAJC1’s involvement in extracellular matrix organization and collagen binding within the GBM TME. DNAJC1 also regulates complement and cytokine signaling, including IL-10, IL-17, and PD-1 pathways, and is associated with EMT. The suppression of DNAJC1 results in decreased EMT marker expression, suggesting its role in promoting EMT and thereby enhancing GBM cell invasion and migration.

Moreover, the infiltration of immune cells into the TME is critical in gliomagenesis (Crivii et al. 2022). Macrophages, particularly those with the M2 phenotype, dominate the inflammatory cell population within tumors and facilitate tumor progression by promoting growth, angiogenesis, migration, and resistance to various therapies (Ross et al. 2021; Mantovani et al. 2017; Gutmann and Kettenmann 2019). Th2 cells and immature neutrophils in the TME contribute to an immunosuppressive milieu that aids tumor immune evasion (Basu et al. 2021; Lin et al. 2022). In our research, we found that elevated DNAJC1 expression is positively associated with the infiltration of macrophages, neutrophils, and Th2 cells into the glioma TME. Inhibition of DNAJC1 led to reduced expression of the M2 macrophage marker CD163. Collectively, these findings support DNAJC1’s role in gliomagenesis through the recruitment of immunosuppressive cells into the TME.

In conclusion, bioinformatic analysis reveals that DNAJC1 exhibits high expression levels in human GBM specimens with a strong correlation to clinical prognostic outcomes. DNAJC1 is implicated in enhancing proliferation, migration, and the recruitment of immunosuppressive macrophages in glioblastoma, and its association with the expression of immunosuppressive molecules, such as CTLA-4, PD-1, and PD-L1, suggests a role in immune cell inhibition and tumor immune evasion. It appears that DNAJC1 may facilitate GBM growth and progression within the TME by supporting immune escape mechanisms and drug resistance, potentially through promoting the epithelial-mesenchymal transition (EMT) process and upregulating immune inhibitory signaling.

Clinically, DNAJC1 holds promise as a prognostic marker and diagnostic biomarker for GBM. However, the complexity and heterogeneity of GBM necessitate further clinical validation and translational research. Personalized therapy, tailored to individual patient subtypes and clinical features, represents an important avenue for future GBM treatment strategies. The combined use of DNAJC1 with other molecular markers and clinical indicators may lead to more customized treatment approaches.

Our study, through a blend of bioinformatic inquiry and clinical data, contributes to a nuanced understanding of DNAJC1’s function in gliomas. Notwithstanding, reliance on public databases could introduce bias, and in vitro loss-of-function experiments may not fully capture the complexity of human gliomas. Consequently, additional research employing animal models is imperative. Overall, our research underscores DNAJC1’s pivotal role in glioma pathogenesis and underscores its potential as a significant prognostic biomarker and a candidate for targeted therapy, opening new pathways for advancing glioma diagnostics and treatment.

### Supplementary Information

Below is the link to the electronic supplementary material.

Supplementary file1 (TIF 61791 KB)

## Data Availability

The original contributions presented in the study are included in the article file, and further inquiries can be directed to the corresponding author.
