# Supplementary figures and images for: DNAJC1 facilitates glioblastoma progression by promoting extracellular matrix reorganization and macrophage infiltration
Source: J Cancer Res Clin Oncol. 2024 Jun 22;150(6):315. doi: 10.1007/s00432-024-05823-1 (PMC11193832; doi:10.1007/s00432-024-05823-1)

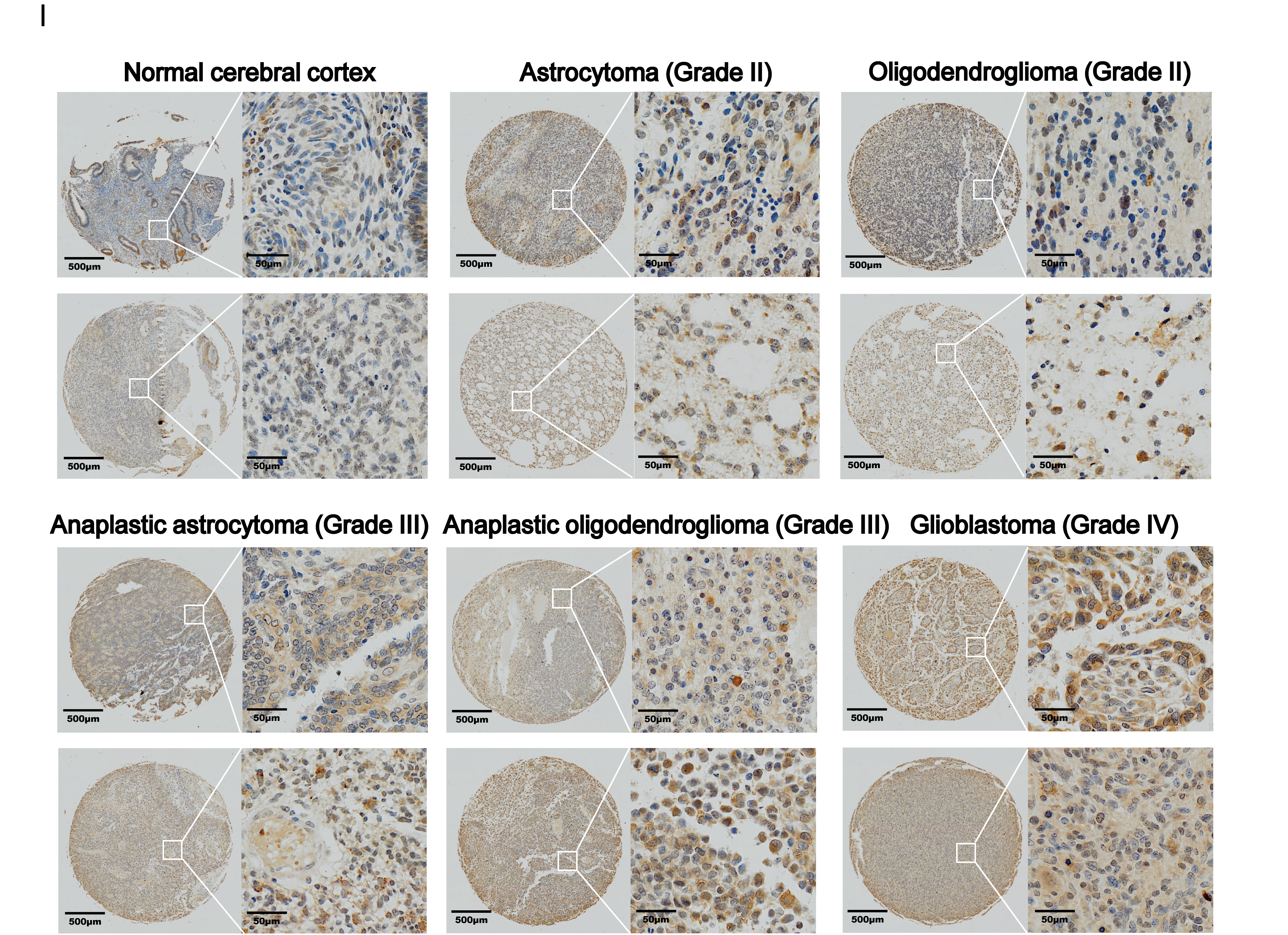

Supplement: Supplementary file 1 — Supplementary file1 (TIF 61791 KB) [file 432_2024_5823_MOESM1_ESM.tif]
